# Magnetic Nanoparticles in the Central Nervous System: Targeting Principles, Applications and Safety Issues

**DOI:** 10.3390/molecules23010009

**Published:** 2017-12-21

**Authors:** Federico D’Agata, Federico Alessandro Ruffinatti, Silvia Boschi, Ilaria Stura, Innocenzo Rainero, Ornella Abollino, Roberta Cavalli, Caterina Guiot

**Affiliations:** 1 Department of Neuroscience, University of Turin, 10124 Turin, Italy; federico.dagata@unito.it (F.D.); federicoalessandro.ruffinatti@unito.it (F.A.R.); silvia.boschi@unito.it (S.B.); innocenzo.rainero@unito.it (I.R.); 2 Department of Neuroscience, Psychology, Drug Research and Child Health (NEUROFARBA), University of Florence, 50139 Florence, Italy; 3 Department of Clinical and Biological Sciences, University of Turin, 10124 Turin, Italy; ilaria.stura@unito.it; 4 Department of Chemistry, University of Turin, 10124 Turin, Italy; ornella.abollino@unito.it; 5 Department of Drug Science and Technology, University of Turin, 10124 Turin, Italy; roberta.cavalli@unito.it

**Keywords:** magnetic nanoparticles, targeting, delivery, central nervous system, blood brain barrier

## Abstract

One of the most challenging goals in pharmacological research is overcoming the Blood Brain Barrier (BBB) to deliver drugs to the Central Nervous System (CNS). The use of physical means, such as steady and alternating magnetic fields to drive nanocarriers with proper magnetic characteristics may prove to be a useful strategy. The present review aims at providing an up-to-date picture of the applications of magnetic-driven nanotheranostics agents to the CNS. Although well consolidated on physical ground, some of the techniques described herein are still under investigation on in vitro or in silico models, while others have already entered in—or are close to—clinical validation. The review provides a concise overview of the physical principles underlying the behavior of magnetic nanoparticles (MNPs) interacting with an external magnetic field. Thereafter we describe the physiological pathways by which a substance can reach the brain from the bloodstream and then we focus on those MNP applications that aim at a nondestructive crossing of the BBB such as static magnetic fields to facilitate the passage of drugs and alternating magnetic fields to increment BBB permeability by magnetic heating. In conclusion, we briefly cite the most notable biomedical applications of MNPs and some relevant remarks about their safety and potential toxicity.

## 1. Introduction

Already in 2006, the World Health Organization (WHO) published a report on brain disorders and their future impact [1]. According to them, brain disorders constitute 12% of total deaths globally, being cerebrovascular diseases responsible for 85% of the deaths due to brain disorders. Together with dementia, such pathologies are strictly related to people aging. As far as dementia is considered, they estimated that, globally, 24.3 million people have dementia today, with 4.6 million new cases annually. Numbers of people affected will double every 20 years to 81.1 million by 2040. Most people with dementia live in developing countries: 60% in 2001 rising to an estimated 71% by 2040.

The economic costs of dementia are enormous, and include “formal care” (health care, social and community care, long-term residential or nursing-home care) and “informal care” (unpaid care by family members, including their lost opportunity to earn income). According to [2] direct costs are about 60% of the total, and the annual European (EU27 + Iceland, Norway and Switzerland) cost for dementia was estimated to be about €105 billion (PPP) in 2010.

Currently there are no treatments to cure dementia. Some drugs (cholinesterase inhibitors), in some cases, but not all, temporarily decelerate the progressive cognitive decline that occurs in Alzheimer’s Disease (AD), and maybe in other forms of neurodegenerative dementia. These drugs act on the symptoms but not on the disease itself; they make only a small contribution to maintaining function.

In the coming next years, innovative approaches in drug delivery to treat Central Nervous System (CNS) diseases are necessary as pathologies such as stroke and Neurodegenerative Diseases (NDs) are becoming an important medical and social issue due to the aging of the general population, and the related economic costs of both therapies and care.

When available, current pharmacological treatment is mainly based on the systemic delivery of active and effective substances, whose effects are severely limited by poor pharmacodynamic properties and intake limitations due to the Blood Brain Barrier (BBB).

Overcoming the BBB and properly deliver the drug cargo in the CNS is therefore the first requirement for an effective therapy, and both chemical and physical approaches have been proposed: See [3,4] for a short summary of the latter.

The present review focuses on the use of magnetic fields in promoting the crossing of the BBB and the driving of the therapeutic agents in the CNS. Current clinical applications, their specific safety issues and future prospects are also briefly outlined.

## 2. Methods

PRISMA guidelines recommendations were followed for the search, the selection and the reporting of the relevant papers (http://www.prisma-statement.org). The following scientific reference resources were used: PubMed and MEDLINE, as we focused on biomedical applications. Data mining was performed through articles published up to October 2017 (time limit), using combinations of the following MeSH keywords: nanoparticles, magnetic, central nervous system, blood brain barrier, and non-MesH keywords: magnetofection, magnetoporation. Searches were limited to articles published in English. We used a query with the following logical combination of the keywords:
nanoparticles AND magnetic AND (central nervous system)ORnanoparticles AND magnetic AND (blood brain barrier)ORmagnetoporation AND (central nervous system)ORmagnetofaction AND (central nervous system).

Cited literature supports the discussion of the role of magnetic targeting/delivery in CNS, but it is not meant to be exhaustive. In our data mining, most of the included research and review articles were published after 2015.

## 3. Results

All the query results were pooled together, removing the duplicates. Resulting references were screened to keep only the papers answering with the following criteria: description of biomedical CNS applications of nanoparticles steered by magnetic force to be targeted on a defined space position or to deliver drugs or therapeutic compounds. Other biomedical applications (e.g., cells destruction by hyperthermia, cells migration/scaffolding) were excluded.

Further references were retrieved manually by reviewing the bibliographies of the relevant publications.

Five hundred and fifty-seven (557) papers were retrieved through database searching and 37 more by looking at the references in the final selection. The number of records selected for the qualitative synthesis was reduced to 76 after the removal of duplicate entries and the selection of relevant papers by reading titles and abstract content.

The selected literature about magnetic targeting/delivery in the CNS was classified into seven main groups:

- reviews [4,5,6,7,8,9,10,11,12,13,14,15,16,17,18,19,20,21];
- in silico modeling [22,23,24,25];
- BBB crossing under Static Magnetic Field (SMF) influence [26,27,28,29,30,31,32,33,34,35,36,37];
- Alternating Magnetic Field (AMF) applications for targeting/delivery [38,39,40,41,42,43];
- magnetofection [44,45,46,47,48,49];
- applications for CNS disease:
  1. brain tumors [50,51,52,53],
  2. neuroAIDS [54,55,56,57,58,59],
  3. wireless Deep Brain Stimulation (DBS) [60,61,62,63,64,65,66,67],
  4. stroke [68,69],
  5. NDs [70,71,72,73,74,75];
- CNS toxicity [76,77,78,79].

## 4. Discussion

### 4.1. General Properties of Magnetic Nanoparticles

In biomedicine, smart drug carrier or smart nanoparticle (NP) design is an evolving technology of outstanding relevance, as their size is comparable with those of cells (<100 μm), viruses (<450 nm), proteins (<100 nm) and genes (length < 100 nm), thus opening the possibility of external manipulation of fundamental biological processes [9]. An important advantage of NPs in improving drug delivery is also their superior bioavailability due to a prolonged presence in the blood circulation and their high surface-to-volume ratio which enhances the contact between the drug and the body fluids [6].

The enhancement of effective drug delivery to the target achieved by the addition of magnetic energy using NPs sensitive to magnetic fields has been widely explored in recent years. The idea is almost 60 years old, since it had already been proposed by Freeman [80] in 1960, but the necessary technology has only recently been developed. Many technical issues still have to be solved before routine application in clinical settings on human patients is possible. Currently, only a very small number of clinical trials are ongoing and very few of them deal with CNS diseases, due to the additional difficulty represented by BBB trespassing [18].

NPs exhibiting magnetic properties are called magnetic nanoparticles (MNPs) and their capability of creating temporary pores in the membranes of the cell—as is the case in the BBB endothelium—to improve targeting and delivery is called magnetoporation [4]. MNPs are already employed in many biomedical applications [4,5,6,7,8,9,10,11,12,13,14,15,16,17,18,19,20,21]:

- magnetic enhancing contrast agents;
- magnetic hyperthermia and heating;
- magnetic labeling and separation;
- magnetic vectors.

As MNPs can be used in both in diagnostic and therapeutic applications, even jointly, they are often considered the prototypical tool for theranostic applications [18]. In particular, drug delivery with the aid of magnetic fields has been termed magnetic drug targeting [6] and several steps have to be pursued to reach this goal [4]: Manufacturing MNPs with a sufficient magnetic coupling, assessing their stability and bioavailability, designing a SMF to push/pull MNPs near a planned location (target), optionally using an AMF or other mechanisms such as ultrasounds [81,82,83] to control/enhance the delivery of the drug.

When systemic (e.g., intravenous) administration is considered, MNP characteristics (size, shape, surface charge, coating, magnetic properties) have to be specifically tailored for the planned clinical application [18]. In particular, MNP optimum size and most performing magnetic properties heavily depend on the physiological characteristics of the target. Simulations [22,23] generally consider three forces acting on MNPs circulating in blood vessels: magnetic drift, diffusion and blood advection. Hence, three regimes are possible:

- magnetic force dominance;
- blood velocity dominance;
- boundary layer formation (when magnetic and drag force are comparable).

Only in this third case, a zone of enhanced concentration of MNPs can be established in a steady state condition at the interface between tissues and blood. If MNPs are retained near the target long enough they may be internalized by the cells. The occurrence of this most favorable regime critically depends on relative values of three non-dimensional parameters:

- the Magnetic-Richardson number, that represents the ratio between magnetic and drag forces;
- the mass-Péclet number, that represents the ratio between advection and diffusion rates;
- the Renkin-reduced diffusion coefficient, that represents the ratio between the diffusivity in the tissue and the total diffusivity in the blood.

Size, shape and coating can alter magnetic forces and resistance by orders of magnitude: exceedingly small particles experience an insufficient magnetic force and too big ones encounter too much resistance in the tissue [24,25]. In general, the field strength should be of the order of 200–700 mT with gradients of about 8–100 T/m, depending on the blood flow (rate ranging from 10 cm/s in arteries to 0.05 cm/s in capillaries) [9]. In the human brain, the working distance (up to 30–50 cm from the magnet surface) and the safety restrictions of the magnetic field strength (8 T adults, 4 T children) limit the applications of MNPs in clinical settings [18]. Magnetic targeting is likely to be more effective for targets near the surface of the body and in regions of slower blood flow [84].

Essentially, many interconnected factors contribute to influence the effectiveness of MNP targeting and delivery [6,9,15,16,19]: the already cited inherent MNP properties—size, magnetic properties, coating, functionalization, biocompatibility (i.e., toxicity)—but also many extrinsic factors—such as the chemical properties of the loaded drugs, the strength, the gradient and the geometry of the magnetic field, the physical (e.g., viscosity) and physiological (e.g., flux) characteristics of the environment in which MNPs move and finally their bioavailability connected to cell uptake and unwanted internalizations.

Moreover, inherent NP properties are also responsible for the stability of their aqueous suspension. MNPs are generally produced as a colloidal ferrofluid: a suspension of magnetic nanoparticles in aqueous fluids with concentrations of about 10^15^–10^17^ particles/mL. Their stability depends on the balance among forces—van der Waals, dipole-dipole, steric, thermal and electrostatic—between the particles and the surrounding solvent molecules. A biocompatible polymeric layer coating (e.g., PEG, polyethylenimine, polylysine, polyacrylic acid) has been demonstrated to improve the stability of MNPs [16]. In contrast, without a proper coating and because of their large hydrophobic surface to volume ratio, MNPs have a greater tendency to aggregate, but on the other hand the large surface-volume ratio provides many chemical sites to conjugate bioactive molecules and functionalize MNPs for many different applications [7,8,12,13,20]. In this regard, special attention to safety has to be paid since the unwanted agglomeration of MNPs can result in blood vessels clotting. An efficient strategy to avoid such a risk is to use superparamagnetic MNPs (see below).

### 4.2. Interplay between MNPs and Magnetic Fields

All materials exhibit magnetic properties that arise from the distribution of electrons spinning around their atomic nucleus. In each atom, nucleus and electrons create a system of interacting magnetic dipoles that cause atoms to act as a permanent dipole provided they do not completely counterbalance because of a symmetric atomic configuration (e.g., iron [9]). A permanent dipole is characterized by its magnetic moment, which represents the torque it experiences when interacting with an external magnetic field. When an external magnetic field (H) is applied to a given material, at microscopic level the atomic dipoles tend to align with the field direction, causing a macroscopic measurable magnetic moment. Magnetization M or magnetic moment per unit volume is proportional to H through a non-dimensional coefficient (χ) called magnetic susceptibility [9]:

(1) $$\vec{M}=\chi \vec{H}$$

If χ is weakly negative (<−10^−3^) the material is diamagnetic, if it is slightly positive (<10^−1^) the material is paramagnetic and if it is largely positive the material is ferromagnetic (>10^3^). For diamagnetic and paramagnetic materials, the relationship between H and M is generally linear, while ferromagnetic materials exhibit some magnetic hysteresis, that is the material could show residual M also after turning off H [9].

In general, magnetization does not increase indefinitely with H due to the limited number of dipoles that can interact constructively under its influence: when this limit is asymptotically approached we get the saturation of the magnetization ($M_{S}$) [9].

Ferromagnetic materials become paramagnetic over a critical temperature called Curie temperature (e.g., 1043 K for iron) and may become superparamagnetic provided their size is lower than a critical value called superparamagnetic diameter [6]. The superparamagnetic diameter has to be smaller than the diameter of the specific material single magnetic domain—in general, less than 50 nm, depending on the material. In this sense, superparamagnetism can be considered as an emergent property of ferromagnetic nanoparticles, where each MNP can be though as the carrier of a single giant magnetic moment, the sum of the individual magnetic moments of the atoms forming it. Normally, these ‘macro-spins’ can randomly flip direction under the influence of temperature and the average magnetization is zero over time. However, in these conditions, an external magnetic field is able to magnetize the nanoparticles, similarly to what happens with a paramagnet, but with χ well higher than the paramagnetic ones [6].

Such properties are satisfied by Iron Oxide NPs (IONs), highly magnetizable MNPs containing iron oxide particles composed of magnetite (Fe_3_O_4_) or maghemite (γ-Fe_2_O_3_). They are often called Superparamagnetic Iron Oxides Nanoparticles (SPIONs) or Ultra-Small Superparamagnetic Iron Oxides Nanoparticles (USPIONs) on the basis of their size (>50 nm or <50 nm, respectively) [33].

Both oxides are ferromagnetic with very similar structures and properties, although magnetite has a larger $M_{S}$ than maghemite (90–100 emu/g vs. 60–80 emu/g), a lower Curie temperature (850 K vs. 948 K) and a lower superparamagnetic diameter (25 nm vs. 30 nm). They both exhibit chemical stability under physiological condition, low toxicity and high magnetic moments.

A variety of methods to synthesize IONs have been described: co-precipitation, thermal decomposition, hydrothermal, solvothermal, sol-gel, microemulsion, ultrasound irradiation and biological procedures. One of the simplest and most commonly employed methods is the co-precipitation of Fe^2+^ and Fe^3+^ from aqueous salt solutions by base addition. The salts used, the Fe^2+^ to Fe^3+^ ratio, pH and ionic strength of the media influence the final size, the shape and the composition of the resulting MNPs [6].

**Figure 1:**
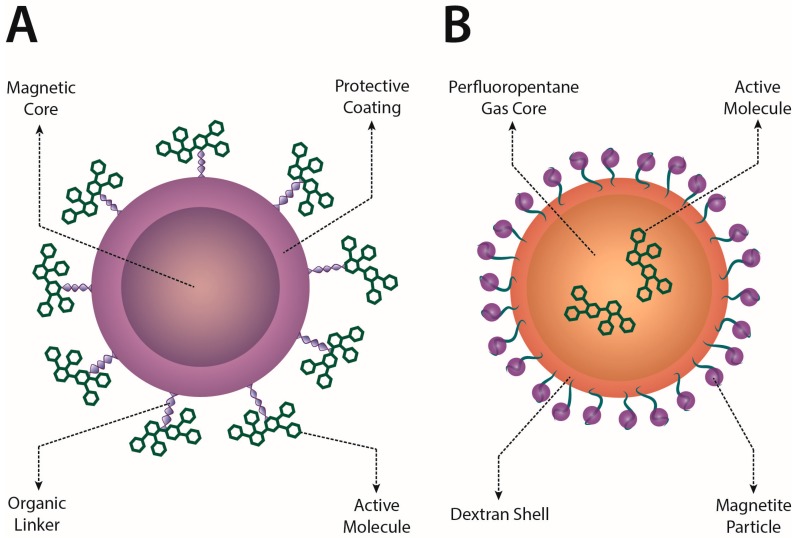
Schematic representation of two strategies for drug delivery through magnetic nano-carriers. (**A**) The magnetic element is the core of the nanoparticles, while the active compound is linked to the protective coating surfacing the core; (**B**) In this case, the magnetic element consists of a number of iron nanoparticles attached to the surface of a nano-bubble structure that can be internally loaded with drug compounds.

IONs can be directly used in targeting application or in combination with other nanovectors, such as polymeric nanodroplets, as those described in Figure 1 and presented in [85].

As already stated, working with superparamagnetic particles is highly recommended as MNPs can respond with a high magnetization, reaching $M_{S}$ under the influence of an external field. Additionally, when the field is turned off MNPs lose their magnetic properties avoiding the risk of reciprocal attraction and agglomeration [9].

IONs are thought to be well tolerated as they gradually are degraded to Fe^3+^ that is integrated into the cellular iron deposits for metabolic processes and its excess can be eventually eliminated [78]. Notably, it is to be paid particular care to the iron concentration in the brain as toxicity and apoptosis could arise due to the great sensitivity of the CNS to iron concentration [76,79].

Using an external magnetic field B acting on a MNPs of volume V, density ρ and magnetic moment M we obtain a magnetic ‘driving force’ [4,7]:

(2) $${\vec{F}}_{M}=\left(\vec{m}\cdot 𝛻\right)\vec{B},$$

(3) $$\vec{m}=\rho V\vec{M}.$$

High static fields are needed to have a high magnetization of the MNPs (up to $M_{S}$) but also high gradients to push/pull them are required. The magnetic force should contrast the drag force $F_{D}$ in the fluid where MNPs are moving with a velocity v (η being the viscosity of the fluid and $r_{H}$ the hydrodynamic radius of the nanoparticle) [4]:

(4) $$F_{D}=6\pi \eta r_{H}v.$$

Hydrodynamic radius $r_{H}$ can be larger (up to 4× or more) than particle radius especially if MNPs have been coated or functionalized [9]. During blood circulation a corona of proteins and other particles could be attracted by MNPs, thus changing their characteristics as bioavailability and interactions with cells [78]. A tradeoff has to be paid for size as small MNPs do not aggregate and more easily cross biological barriers, but they respond less to magnetic force (see Equations (2)–(4) $F_{M}\;\sim \;r^{3}$ vs. $F_{D}\;\sim \;r$). In any case, superparamagnetic radius gives a superior limit to ION size.

Although in principle magnetic targeting should be feasible with any accuracy, currently there are no systems capable of a precise deep focusing. A general limit for the creation of a magnetic static trap had already been demonstrated by Samuel Earnshaw in 1839. Relative focusing is possible only using dynamic magnetic fields [18]. Moreover, B rapidly decreases when penetrating living tissues, thus dramatically affecting the efficiency of the delivery to deep targets, located far away from the surface of the body. As a result, even if several in vitro and animal studies have been reported, clinical applications are still very challenging due to difficult targeting in deep tissues (namely >2 cm depth) and because particles exhibit poor retention when the external magnetic field is turned off [9].

Summarizing, drugs encapsulated or attached to MNPs are relatively simple to synthetize, penetrate easily in living tissues, have a low toxicity (good biocompatibility) and can be remotely controlled by external non-invasive magnetic fields. With particular reference to the SNC, the advantages of magnetic drug targeting in the brain are: noninvasiveness, no direct contact, efficient control of drug delivery; cons are: possible accumulation and cytotoxicity, possible aggregation and embolization, limited bioavailability to brain due to absorption by other organs (liver, kidneys), fast decay of the magnetic field from surface.

Some of the downsides could be turned to advantages, for example enhanced toxicity against tumor cells or closing of the blood vessels by clotting if needed (e.g., arteriovenous malformation, hemorrhages).

### 4.3. BBB: Critical Issues and Trespassing by MNPs

**Figure 2:**
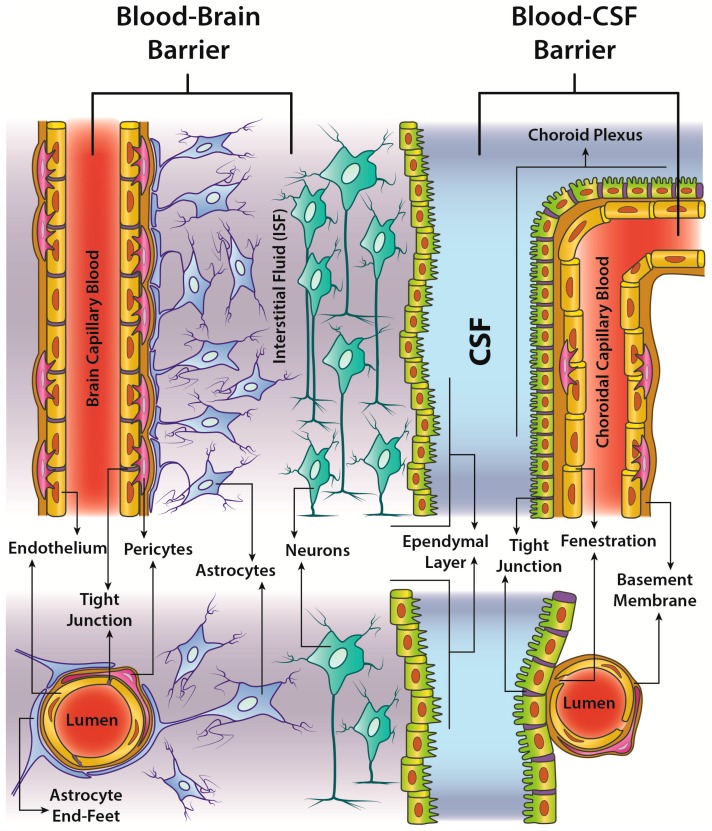
Comparison between the structure of the Blood-Brain Barrier (BBB) and the blood-CSF barrier. (**Left**) BBB separates the lumen of the brain capillaries from the brain parenchyma. The main contribution to the BBB property of reduced permeability comes from the tight junctions (drawn in violet) among endothelial cells lining the capillaries. The so-called neurovascular unit also comprises the pericytes, a basement membrane surrounding both pericytes and endothelial cells and astrocyte end-feet processes from nearby astrocytes. As well as the undisputed role of the tight junctions in sealing the interendothelial cleft, all the elements of the neurovascular unit are likely to contribute to some extent to the augmented selectivity of the BBB. That said, their role is still controversial; (**Right**) The Blood-CSF barrier is found in the choroid plexus of each ventricle of the brain. Unlike the endothelium in the brain parenchyma, capillaries of the choroid plexus have no tight junctions and are fenestrated. However, the choroid plexus is delimited overall by a monolayer of tight-junctioned epithelial cells. This particular epithelium is in direct continuity with the ependymal layer lining the ventricle, though the rest of the ependymal layer is much more permeable. Therefore, unlike the BBB, the blood-CSF barrier is located at epithelial level, while capillaries are relatively leaky and permeable to small molecules, thus allowing, among other processes, the rapid delivery of water through the bloodstream to the surrounding epithelial cells for CSF production in the choroid plexus. Similarly, to what can be found in other tissues of the body, also in the choroid plexus pericytes and a basement membrane wrap around the endothelial cells. Although in principle both the barriers serve the same defensive purpose for the CNS, their distinct structure allows the interchange of different substances between bloodstream and brain. (**Upper part**) Top view; (**Lower part**) Section view.

Despite the extended surface area of about 20 m^2^ of the 100 billion capillaries feeding the human brain [6], many substances have very limited diffusion from blood to neurons because of the existence of the BBB and blood-Cerebro Spinal Fluid (CSF) barrier (Figure 2).

The endothelial cell layer of the capillaries of the brain parenchyma has features which are unique in the human body, in that it presents tight junctions between adjacent cells and no fenestrations. This causes an increased overall Trans-Endothelial Electrical Resistance (TEER), as high as 2000 Ω·cm^2^ [9]. Moreover, brain endothelial cells have a reduced transcellular general permeability (scarce endocytotic vesicles) and many ATP proteins (e.g., P-glycoprotein and multidrug resistance protein) that act as efflux pumps keeping in blood circulation most of the molecules. As a consequence, up to the 98% of the substances—including therapeutic agents—cannot reach the brain [6].

**Figure 3:**
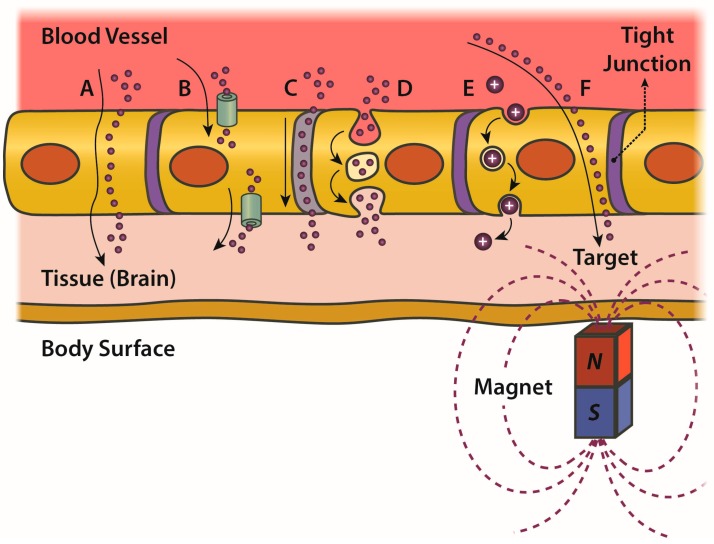
Schematic enumeration of the many pathways which a compound can use to cross the blood-brain barrier, depending on its chemico-physical properties. (**A**) Drugs can cross the barrier simply by passive diffusion if they are sufficiently lipid soluble (or have been made lipophilic by appropriate chemical modifications); (**B**) Carrier protein-Mediated active Transport (CMT) can allow many essential compounds such as glucose and amino acids to enter the endothelial cytoplasm and then be released into the brain at the abluminal side. In addition, artificial compounds mimicking those endogenous ligands have been developed to take advantage of the carrier-mediated transport mechanisms; (**C**) Drugs or even NPs can enter the brain through a paracellular route only when the tight junction system is disrupted. In particular, BBB permeability can be temporarily induced in several ways: by local temperature increases (38–39 °C), by osmotic alterations (e.g., infusion of hypertonic solutions of mannitol), by adenosine receptor activation, by Focused Ultrasound (FUS) bombardment or by electromagnetic radiations; (**D**) The binding of specific ligands to the receptors mediating the endocytosis (e.g., transferrin and insulin receptors) allows the uptake of large compounds and NPs conveniently functionalized. By a subsequent exocytosis process, vesicles can then release their content at the abluminal side (RMT); (**E**) If the compound has sufficient cationic charge, it can induce a localized electrostatic disruption of membrane phospholipids resulting in the so-called Adsorptive-Mediated Transcytosis (AMT); (**F**) If the drug is carried by NPs exhibiting magnetic properties (such as IONs) a localized magnetic field, generated by an external electromagnet, can be used to produce a driving force enabling the passage of such NPs from the bloodstream to a targeted region of the brain (through both paracellular and transcellular routes).

This highly efficient defense mechanism allows exceptions only for those elements needed for brain functioning (e.g., glucose, amino acids) admitting four main ways [76] to trespass the barrier (Figure 3):

- passive transcellular diffusion (only O_2_, CO_2_ and small lipid-soluble substances),
- Carrier protein-Mediated Transport (CMT),
- Receptor-Mediated Transcytosis (RMT),
- Adsorptive-Mediated Transcytosis (AMT).

RMT generally works for large peptides and proteins (>600 Da); examples are the processes of endocytosis triggered by insulin and transferrin receptors [6]. CMT generally allows smaller nutrient molecules (<500 Da) crossing the barrier; examples are the transporter for glucose, amino acids, nucleosides and choline. AMT takes place when a positively charged compound is able to induce a pronounced and localized disruption of plasma-membrane integrity; examples are cationized albumin or synthetic NPs coated with molecules such as chitosan.

Notably, to date, the only drugs actually capable of crossing the BBB in therapeutic concentrations are small lipophilic molecules (<500 Da) used in the treatment of CNS diseases, especially in psychiatric disorders. Many other potentially useful drugs cannot be used unless new strategies to overcome the BBB are devised, possibly exploiting the physiological mechanisms previously listed (in particular CMT and RMT) by coatings able to mimic the ligand-protein interactions.

In addition, a number of non-invasive and effective strategies to loosen tight junctions and thus induce a transient increase in BBB permeability have been recently developed, possibly enabling macromolecules to enter the CNS via paracellular route. In particular, Focused Ultrasound (FU) used to induce mechanical stress at cellular level can temporarily disrupt tight junctions and represent one the most promising approach. In this regard, at the Sunnybrook Research Institute (Toronto, ON, Canada), O’Reilly and colleagues have employed low-frequency FUS to safely open the BBB in half of the brain of aged dogs [86]. They succeeded in increasing BBB’s permeability by about 20% with no evidence of tissue damage in all animals tested.

Temporary osmotic opening of BBB (e.g., by mannitol infusion [32]) is another proven technique to increase BBB permeability. Although this method has been controversial for decades because of the variability of its outcomes, recent developments that take advantage of arterial microcatheterization and MRI guidance techniques have made it super-selective, more reliable and predictable [87].

Moreover, adenosine-mediated BBB modulation is another recent and notable approach: Adenosine receptor activation can indeed increase BBB permeability, while the antagonism of its signaling has been reported to tighten junctions between endothelial cells in both in vivo and in vitro models [88].

Other possibilities to bypass the barrier include the intranasal administration [89] or the opportunistic delivery, when taking advantage of particular situations, such as tumors, inflammatory and infectious sites where the BBB permeability is enhanced as consequence of the leaky vasculature of the affected tissues.

In the sections below, we will focus on those MNP applications allowing a nondestructive BBB crossing (see Table 1): Essentially the use of magnetic fields to facilitate the passage of drugs by mechanical-driven magnetic carriers or to increment BBB permeability by magnetic heating.

### 4.4. In Vitro and In Vivo Evidence of BBB Trespassing by MNPs under Static Magnetic Fields

In vitro studies were mainly performed by administrating MNPs of different size and characteristics to in vitro BBB models obtained by co-culturing endothelial cells and astrocytes in the presence of static magnetic fields.

In [33] Brain Capillary Endothelial Cells (BCECs) were first stained for the tight junction protein ZO-1 and then incubated with IONs at different doses (35, 70 and 140 µg/mL) for 24 h, showing that internalization and transcellular transport occurred also in the absence of magnetic fields and without exerting any toxic effect. By adding a magnetic force produced by a ferrite block magnet and corresponding to B = 0.39 T, the BCEC monolayer did not lose its integrity (the initial TEER = 43.2 ± 0.5 Ω·cm^2^ was unchanged after 5 h incubation with IONs and also after 5 h exposure to B). The passage of IONs across the BCEC monolayer was concentration-dependent and was significantly increased by B at all concentrations (11-fold at 35 µg/mL, 8-fold at 70 µg/mL and 29-fold at 140 µg/mL). Finally, after having crossed the endothelial monolayer, nanoparticles entered the ‘brain side’ of the barrier and fluorescent IONs could be detected in the co-cultured astrocytes, even at remote distance.

Therefore, IONs proved able to:

- pass the BBB in the field direction,
- not modify the TEER (i.e., no damages to the barrier integrity),
- not alter the cell viability,
- be internalized by astrocytes.

Similar conclusions have been reported by different groups using in vitro BBB models with different MNP formulations and magnetic field intensities [26,30]. Another interesting application of magnetic driving has been proposed in [34] for intrathecal drug targeting in the CNS: here Venugopal and colleagues used SMF to target gold-coated magnetite NPs to specific regions along the spine, employing both in vitro and in vivo models. The gold coating served to prevent oxidation and assure the biocompatibility of the MNPs, whose overall diameter was between 25 and 40 nm. First, a human spine phantom was prepared, to simulate the administration on MNPs in the CSF of the subarachnoid space under magnetic driving. The optimal settings for the magnet placement and field strength were investigated in order to drive the MNPs at specific spinal levels. Experiments were then repeated on Sprague-Dawley rats, performing the injection of 20 µL solution of MNPs (17 µg/mL) in the CSF between L4 and L5 vertebrae and placing a small magnet (0.01 T) in different positions between T9 and T1 vertebrae. Animals were imaged with 9.4 T MRI and later by histology, showing that magnetic steering was very effective in localizing the MNPs in specific regions of the spine, without any evidence of toxicity. The same group has proposed an implant-assisted intra-thecal magnetic drug targeting system [29]. Promising results in MNP targeting have been reported also in the brain cortex of the same animals and in other animal models as well [27,28,30,31,35,36,37].

### 4.5. Heating of MNPs by AMF: Applications for Increasing BBB Permeability and Drug Delivery

The magnetic moment of a MNP can only be oriented along two stable directions antiparallel to each other and separated by an energy gap ΔE that for IONs is comparable to thermal energy. In the absence of any external magnetic field, this results in a total magnetization that fluctuates randomly. On the contrary, the application of an external AMF to MNPs reorients their magnetic moments and produces heat. 

In multidomain ferromagnetic materials the production of heat is due to hysteresis losses; in single domain materials, as superparamagnetic IONs, the external magnetic field causes the rotation of the magnetic moments, but this excitation is rapidly dissipated when the particle moments relax to their equilibrium orientations. The Néel relaxation time $t_{N}$ associated to this phenomenon is:

(5) $$t_{N}=t_{0}e^{\frac{\Delta E}{kT}},$$

where $t_{0}$ (whose value typically ranges between 10^−9^ and 10^−10^ s depending on the material) is the so-called *attempt time*, T is the absolute temperature and k is the Boltzmann constant.

Another heating mechanism is possibly due to the rotational Brownian motion that creates frictional losses into the surrounding environment. The Brown relaxation time is:

(6) $$t_{B}=\frac{\eta \pi r_{H}^{3}}{4kT}.$$

A general effective time of relaxation $t_{eff}$ comes from the combination of (5) and (6):

(7) $$\frac{1}{t_{eff}}=\frac{1}{t_{N}}+\frac{1}{t_{B}}.$$

which phenomenon dominates depends on size, viscosity and magnetic properties of the materials. In general, for IONs, Néel relaxation predominates.

As a consequence, IONs can heat in presence of an AMF (up to 45 °C) and can be employed for thermotherapy or for inducing a reversible increase in BBB permeability, involving both the paracellular pathway (loosening of BBB’s tight junctions) and the transcellular one. At high AMF frequencies, the heat generated is such (reaching *t* > 42 °C) as to damage cells and tissues, while the heat produced by low frequencies is enough (*t* ~ 39 °C) to enhance BBB permeability without perturbing other brain cells [6].

Such effect was monitored in vitro by Dan et al. [38] (bEnd.3 and MDCK-II cell lines, seeded on Transwell filters and exposed to a 33.4 kA/m AMF at a frequency of 300 kHz) and in vivo by Tabatabaei et al. [43] (using Evans Blue fluorescent dye to test the permeability of brain capillaries in rats induced by a 7.6 kA/m field at 150 kHz).

One of the main goals of this approach is obviously that of overcoming the BBB for loading drugs to CNS. In this regard, Do and colleagues [71] investigated the guidance of magnetic nanocontainers that can potentially carry therapeutic substances across the BBB (specifically fluorescent carboxyl magnetic particles, 700–900 nm in diameter) using both direct current (DC) and alternate current (AC) electromagnetic driving systems. A two-coil electromagnetic actuator was developed to steer the MNPs in the desired direction operating on the input current (between 1 and 3 A) and produce a magnetic field ranging between 28.1 and 79.8 mT with gradients between 0.43 and 1.39 T/m. Mice were injected with 0.4 mL of saline solution containing fluorescent MNPs and were treated without or with DC and AC magnetic fields of different strength for 1 min. Brain tissue was then sliced and evaluated with confocal microscopy images, showing that there was no significant accumulation of MNPs in the absence of magnetic field. The uptake was increased in DC configuration, but higher magnetic forces corresponded to an increased aggregation of the particles. Under AC conditions not only the trans-BBB transport was larger than at the corresponding DC values, but MNP aggregation was avoided.

This result may be of interest for optimizing the processes of electroporation, e.g., for gene delivery or anti-HIV drug applications. Similarly, in [41] nanoelectroporation of therapeutic cargoes was investigated using magneto-electric nanoparticles made of BaTiO_3_ and CoFe_2_O_4_, with an average size of 25 nm, which showed to be biocompatible on microglia cultured on a gold chip, at doses <50 µg for 10^6^ cells. Particle accumulation in microglia was observed when an AC-magnetic field of strength ranging between 40 and 80 Oe was applied. Interestingly, different phenomena were observed at a range of field strengths, i.e., a more superficial accumulation at lower field, which tended to interest the deeper layers when field intensity was increased (although also toxicity due to heat generation was greater).

Finally, the heating of MNPs can also be used as an externally controlled trigger of the loaded drug release. The thermal energy can be used indeed to open the gates for any kind of organic or inorganic carriers (e.g., thermoresponsive nanogels) and AMF can provide fully reversible and controllable transmission of this energy [9]. In particular, AMF have been demonstrated to be capable of an accurate time control of the release of different drugs in different in vitro models [39,40,42].

### 4.6. Magnetofection and CNS

Magnetofection is defined as the delivery of genetic material (with viral or non-viral vectors), usually performed in vitro by means of an high-field/high-gradient magnet placed underneath the well culture plate where the cells are growing. The in vivo applicability of the technique is still uncertain.

Magnetofection shows significant advantages over traditional transfection methods (up to 50× increase in transfection levels), including reduced process time (10 min vs. 2–4 h), higher success rates with lower vector doses, preservation of the cells membranes with high post-transfection viability [9].

The method was developed by Christian Plank and collaborators [90] for gene transfer in cell cultures and in vivo using MNPs or MNP-viral complexes conjugated with DNA.

Improvements have been observed by using oscillating magnet field under the plate or perpendicular to the magnetization vector. This mechanical stimulation promotes more efficient endocytosis and increases transfection efficiency [44].

Neurons are particularly sensitive to cytotoxicity and generally difficult to transfect, there is therefore a growing interest in developing MNP formulations and magnetofection protocols suitable for neuronal cell cultures [45,46,48]. Gene transfer to the CNS poses significant challenges due to both the relative inaccessibility of the brain and the extraordinary complexity of CNS structures. On the other hand, this approach offers unique advantages for the long-term delivery of neurotrophic factors to specific CNS regions.

The technology for magnetic field-assisted gene delivery has now advanced to a point from where it seems feasible to implement minimally invasive gene therapy strategies for the brain in vivo [16,47,49].

### 4.7. MNP Biomedical Applications

MNP targeted administration showed promising results in many different CNS diseases (see Table 2). For instance, in brain tumors, they enhanced chemotherapy efficacy [53], and were found to be useful to deliver gene therapy [51] or as nanosurgeons using mechanical force to destroy malignant cells [50,52].

In particular, Chertok et al. used 110 nm nanoparticles with an iron oxide core and a starch coating to target a brain tumor in an in vivo model of glioma (rats harboring orthotopic 9 l-gliosarcomas) [91]. In this work, they showed that magnetic targeting significantly enhanced the accumulation of MNPs in gliosarcomas. Interestingly, in that case, the problem of passing the BBB was partially mitigated since the glioma typically implies structural endothelial abnormalities that make tumor vasculature more leaky and permeable. Notably, the same pathological alterations in brain tumor microcirculation could be used to discriminate the diseased site from healthy brain [84].

Wang et al. [92] successfully co-loaded magnetic liposomes with both drug and gene to treat glioblastoma in a rat model. This multifunctional approach to nanocarriers is of particular interest because it represents a first step towards the design of a new class of “smart” MNPs (see Section 5).

MNPs can be helpful also in anti-HIV therapy. Being the brain one of the main sources of virus production to the periphery, a low delivery of anti-retroviral drugs across the BBB prevents the complete eradication of HIV from the body. Magnetic nanocarriers can be used to target hidden latent virus in the brain, but also to treat the neurological disorders caused by HIV damage to the CNS (neuroAIDS), consisting in neurocognitive impairment and HIV-associated dementia. Specifically, nanocarriers were used in vitro for delivering anti-HIV drugs to reduce infection levels and oxidative stress, but also to regulate synaptic activity and recover spine density [54,55,56,57,58,59].

By introducing heat-sensitive capsaicin receptors into nerve cells and then injecting MNPs into specific brain regions it is possible to control the activation of specific neural activity [60,65] or gene expression evoking specific behaviors in animal models, provided AFM is applied [62,63]. This opens to the possibility to develop a wireless Deep Brain Stimulation (DBS) [64,67], an application potentially valuable for many CNS diseases (e.g., NDs). Notably, a successful modulation of brain activity in mice by injecting MNPs into the blood circulation and forcing them to cross the BBB has also been reported [61].

Moreover, MNPs can be used in stroke to deliver neural stem cells [68,69], and can offer a new strategy for drug delivery in NDs [75] such as Alzheimer’s [70,71,72] or Parkinson’s disease [73,74]. In particular, by using pulsed magnetic fields, Amin and coworkers [70] succeeded in drive dextran-coated Fe_3_O_4_ MNPs loaded with osmotin to the brains of Aβ_1–42_-treated mice. After osmotin delivery, they measured an attenuation of Aβ_1–42_-induced synaptic deficits, Aβ accumulation, tau hyperphosphorylation, and BACE-1 expression.

The use of magnetic resonance has been proposed to drive and image in real time MNPs in deep tissues trying to maximize the target focusing, since the high magnetic field (>1.5 T) allows IONs to achieve magnetization saturation near deep targets [9]. However, traditional MRI scanners are not optimized for particle driving and need to be customized or, at least, upgraded with additional steering coils [93,94]. As a result, while this approach has been essentially presented as a proof-of-concept application, research is still ongoing to create a new effective platform being capable of interleaving imaging and targeting to enable real-time image-guidance of MNPs [18]. In general, while precision feedback control of a single magnetic particle has already been demonstrated in animals and in patients, the gathering of a large number of MNPs in a small and precise area of the CNS has proved to be more difficult [18].

Magnetic Particle Imaging (MPI) is an innovative technique which uses the nonlinear magnetic response of MNPs under strong (>3 T/m) magnetic field gradients. MPI has the potential of imaging MNPs with high spatial and temporal resolution. In particular, the spatial resolution of MPI (and also of MRI) could be improved with a dedicated hardware (currently still under development) able to create ultra-fast magnetic pulses (e.g., with rise times of less than 10 µs) that can overcome the effect of peripheral nerve stimulation [18].

Animal and in silico modeling should be used together to design and project the most appropriate MNPs for different clinical indications. In general, an in vivo real-time imaging and targeting of MNPs is the major goal to be pursued [18].

### 4.8. Safety and Toxicity of MNPs

Conflicting results have been reported for NP toxicity and more accurate studies are mandatory [76], especially in the context of MNPs and IONs, where the chronic effects have received low attention [78] up to now.

NPs are generally eliminated by metabolic pathways and a large fraction of them is captured by the reticuloendothelial system (monocytes and macrophages), but metal NPs have been shown to be particularly critical for brain and BBB as they can induce neurotoxicity, neuroinflammation and permanently alter the BBB permeability [76].

In particular, iron homeostasis is an important process both at a general body level and is involved in DNA synthesis, mitochondrial respiration, oxygen transport. It is also involved at the CNS level in metabolic processes, myelin synthesis and neurotransmitter synthesis. ION administration could interact and possibly alter these processes [79] causing the upregulation of proteins associated to the cellular iron transport and storage and the downregulation of proteins associated to the cellular iron uptake. Iron accumulation could change the cellular equilibrium producing more Reactive Oxygen Species (ROS) via Fenton’s reaction [79]. ROS would in turn cause oxidative stress promoting protein aggregation (e.g., Aβ in Alzheimer’s disease and α-synuclein in Parkinson’s disease) that can contribute to neurodegeneration. CNS is particularly vulnerable to ROS and oxidative stress since there is less enzymatic activity against free radicals than in the rest of the body; moreover a high iron level could inhibit the occludin expression, a protein essential for tight junction integrity, thus leading to a BBB damage [79]. Iron accumulation and neurotoxicity critically depend on the MNP characteristics, in particular, their persistence in the system, which is generally appreciated for the improved pharmacodynamics properties, but could also worsen the toxic effects [79]. The surface charge is another important characteristic as high concentration of anionic or cationic NPs can affect the neurovascular unit compromising the BBB effectiveness [76]. In particular, ION uptake from the glial cells seems to be larger if compared to other CNS cells so they could be more sensitive to the toxic effects [79].

Degradation by macrophages is very slow and iron is metabolized over 6–12 weeks [77] in a dose-dependent manner, but partially retained in the epithelial cells of the choroid plexus and in the neuroglial cells [77]. When neuronal cells are exposed to IONs, however, some adaptation to local iron content occurs by downregulating the correspondent receptors [79]. Favorable properties were also detected: the formation of a protein corona around the NPs [77], able to alter their chemico-physical properties and improve the transport across the BBB [76] and the uptake by monocytes was of special interest.

Finally, it is worth reporting that some ION-based contrast agents had already been developed in the 1990s [95,96] and the adverse effects connected with their administration are well described in literature. More recently, in the USA the marketed anti-anemic drug ferumoxytol (Feraheme™, AMAG Pharmaceuticals), composed by IONs with strong T1 and T2 relaxivities, has been used off-label for MR imaging, while in Europe a similar compound (ferumoxtran-10 Sinerem^®^ (in the EU, Guerbet S.L.)/Combidex^®^ (in the US, AMAG Pharmaceuticals)) is gaining popularity among a new generation of radiologists [77]. Such clinical experiences shed new light on the secondary toxic effects, fully reported by [77]. In addition to the severe anaphylactic reactions to intravenously administered ferumoxytol NPs, also immune responses of the Gell-Coombs system, non-immune reactions (such as the complement activation-related pseudoallergy) and long-lasting skin discoloration were reported [77].

## 5. Challenges and Future Perspectives

As reported in the previous sections, there are numerous papers in the recent scientific literature reporting successes in the field of magnetic targeting and of MNP-mediated BBB crossing and drug delivery, but none go beyond the proof-of-concept level. In the past decades, MNPs (especially IONs) have been generally regarded as one of the most promising theranostic tools, but in the case of biomedical applications, it should be noted that, until now, most of the in vivo studies have been restricted to animal models (and therefore to small organisms) and there are very few clinical trials. Moreover, to date, no commercial applications of magnetic targeting in human can be found and the only truly marketed clinical application of MNPs consisted of IONs used as enhanced contrast agents in the field of NMR (Feridex^®^ (in the US, Berlex Laboratories)/Endorem™ (in the EU, Guerbet S.L.), Resovist^®^ (in the EU and Japan, Bayer Healhcare)/Cliavist™ (in the EU), Sinerem^®^/Combidex^®^, etc.).

The main challenge in this field appears to be that of moving from the lab environment to the clinical setting. More into details, the precise and deep focusing of magnetic fields for the controlled targeting of drug-releasing nanocarriers in human tissue should be pursued (see e.g., [70]). Some of the reasons that make this goal so difficult are essential and specific technical sub-challenges and are here briefly summarized.

The first hurdle is the fact that the field strength rapidly diminishes with target depth in the body. This problem is of particular relevance when moving from small animal in vivo studies to human clinical applications [9,18].

Earnshaw’s theorem (see Section 4.2), which establishes the impossibility of creating a magnetic trap just by using static magnetic fields, is the second limitation. For this reason, magnetic focusing systems need to be though in terms of dynamic fields and their space- and time-averaged values.

The third major complication is the need of driving a large number of magnetic objects to get any measurable therapeutic result. If it is proved that the single MNP can be effectively targeted with high precision and a good feedback control [18], the use of dynamic fields affects the focalization capability when dealing with a large number of MNPs. This is due to the fact that while one particle is driven toward its target, the same (alternating) magnetic field may drive another particle away from the target.

Another challenge in the clinical use of MNPs is their poor retention upon removal of the external magnetic field, thus severely impinging on particle/drug uptake [9]. In this regard, searching for some strategy to speed up the internalization processes seems to be key to avoid more invasive alternatives (e.g., surgical implantation of a magnet in the proximity of the body district of interest). The need to control the uptake rate and the local availability of injected MNPs is even greater when the target is within the CNS, because of the presence of the BBB.

In addition, despite the many above mentioned promising techniques allowing to increase the BBB permeability—and the proved capability of steering MNPs across the barrier—the search for increasingly effective and safe methodologies to control its selectivity and ensure its recovery after drug delivery remains a crucial issue from a clinical point of view.

A possibly less technical but more cultural challenge will possibly be the willingness to create collaborative networks on specific topics within the wide scope of MNP biomedical applications. Due to the intrinsically interdisciplinary nature of this field, only by combining expertise coming from different disciplines, according to a real translational approach, will allow to achieve the main goal stated above. Starting from a specific disease, a taskforce including physicists, chemists, biologists, pharmacologists, clinicians and engineers, integrating their knowledge and combining their efforts, should be able to determine, among a virtually infinite number of combinations, the optimal parameter values to solve that particular problem, that is to choose the best MNP type, dosage, functionalization, drug cargo, driving/delivery hardware system, BBB crossing techniques, also accounting for the bioavailability of the nanoparticles and the minimization of their toxicity.

This integrated approach is also the key to understand what is coming on. A new generation of scanners able to implement a real-time imaging of MNPs interleaved with a strong external driving field for steering them at the same time is likely to be soon available [18]. In addition, integrated hardware devices dedicated to magnetic heating or FUS generation for BBB trespassing could accompany these new all-in-one theranostic platforms.

Finally, new smart magnetic nanocarriers, combining different technologies and specifically thought for CNS applications, are likely to be designed [92]. Their surface could be functionalized with special molecules (or antibodies) able to bind specific receptors located at the luminal side of brain capillaries in order to trigger transcytosis, while their core could be filled with genetic material or multiple drugs that will be released only upon BBB crossing.

## 6. Conclusions

The peculiar properties of MNPs make them ideal for the physical targeting of CNS to optimize drug delivery. In general, only small-sized NPs characterized by stringent surface properties can cross the BBB. However, only a minority of the NPs developed so far has proven to be able to reach the brain from the bloodstream. So, despite the fact that the study of the BBB—and the related search for innovative methods to cross it—have been areas of intense research for many years, we are still far from easily and safely controlling its permeability. For this reason, alternative strategies have been adopted to bypass or temporarily disrupt the BBB. The magnetic driving via external steady or alternate magnetic field has been explored as well and seems to be very promising for future clinical applications.

## Figures and Tables

**Table 1:** MNPs Blood Brain Barrier applications.

IONs	MNPs	Magnetic	Mechanism	Model	Effect	Preparation	Toxicity	Ref.
Fe_3_O_4_	PEGylated fluorescent liposomes + Transferrin Diameter = 130 nm	Static 0.08 T 24 h	Transferrin (RMT) + magnetic force promote crossing	In vitro BBB human endothelium + astrocytes	+50–100% transmigration 2 pg Fe/cell uptake	Coprecipitation aqueous 2 h stability	TEER and cell viability unchanged at 48 h	[26]
Fe_3_O_4_	Polysorbate 80 Diameter = 11 nm (hydro 29 nm) ζ = 19 mV	Static 0.3 T 2 h	Poly adsorb protein (RMT) + magnetic force promote crossing	In vivo rat BBB	Accumulation in near cortex 0.6 mg Fe/g tissue uptake 9-fold increase	Mixing 0.2 g Tween 80 with 0.4 PEG IONs	Cell viability unchanged at 72 h	[27]
Fe_3_O_4_	Silica-coated nanocapsule Diameter = 100–150 nm *M_s_* = 5-fold SPION	Static 1000 Oe 1 week RF (100 MHz)	Cell membrane translocation	In vivo mice BBB	25-fold increased concentration	Emulsion polymerization	Slight reversible astrogliosis No immunotoxicity	[28]
Fe_3_O_4_	Gold coated Diameter = 25–40 nm *M_s_* = 30 emu/g	Static 0.01 T 6 h	Accumulation by magnetic force	In vitro human CSFBB + in vivo rat CSFBB	Up to 50% MRI signal difference confirmed local histological accumulation	Coprecipitation aqueous	High cell viability	[29,34]
Fe_3_O_4_	BDNF binded Diameter = 60 nm	Weak static magnet exposition	Magnetic force	In vitro BBB human endothelium + astrocytes	73% BDNF cross 3.5-fold increase Suppress apoptosis Spine loss reversed	Coprecipitation	High cell viability and TEER unchanged	[30]
Fe_2_O_3_	Oleic acid coated Diameter = 220–250 nm ζ = −4 to −17 mV	Static 8000 Gauss	Passive diffusion or RMT + magnetic force	In vivo rats	Indocyanine green load increased brain concentration 5% tot dose	Thermal decomposition	-	[31]
Fe_3_O_4_	Aminosilane or EDT coating Diameter = hydro 25 or 29 nm ζ = 21 or −39 mV	Static 0.06–0.1 T 24 h	Improve concentration after mannitol opening BBB	In vitro mice endothelium	Flux increase to 44% for EDT after osmotic opening	Aqueous phase reduction/hydrolysis	No change in permeability	[32]
Fe_3_O_4_	Lipophilic fluorescence dye covered by α-d-glucose units Ms = 350 kA/m Diameter = hydro 117 nm ζ = −17 mV	Static 5 h	Magnetic force	In vitro BBB human endothelium + rat astroglia	11, 8, and 29 fold uptake increase of 35, 70, and 140 μg/mL	-	TEER and cell viability unchanged at 29 h	[33]
Fe_3_O_4_	Amphotericin B magnetic liposomes Diameter = 240 nm *M_s_* = 32 memu/g	Static	Magnetic force	In vivo rats	Histological accumulation 400 ng/g brain (after 30 min)	Film dispersion–ultrasonication	Reduced death with magnetic field	[35]
Fe_3_O_4_	Cationic polymeric liposome Diameter = 20 nm	Static 0.5 T	Magnetic force	In vivo rats	Paclitaxel concentration 3-fold histological accumulation	Thin-layer evaporation	-	[36]
Fe_3_O_4_	SiO_2_-coated+Amino Tat peptide Diameter = 100 nm ζ = 42 mV *M_s_* = 19 emu/g	Static 2 h	Magnetic force + transport Tat	In vitro BBB human endothelium + glioma	Cell internalization 2.6-fold increase Permeability 2.3-fold increase	Alkaline co-precipitation	TEER moderate decrease High cell viability	[37]
Fe_3_O_4_	Cross-linked poly(ethylene glycol)-poly(aspartate) or citrate-coated Diameter = 25 nm or 90 nm	Alternate 33.4 kA/m at 300 kHz	Temperature opening BBB	In vitro mice or dogs	2–3 -fold flux increase 3-fold cell uptake increase	Co-precipitation	No cell death	[38]
Fe_3_O_4_	Poly(maleic acid-*co*-olefin) coated Diameter = 11–13 nm	Alternate 7.6 kA/m at 150 kHz	Temperature opening BBB	In vivo rats	Histological accumulation only after RF	-	Reversibility of opening No brain immune response	[43]

**Table 2:** MNPs biomedical applications.

MNPs	Magnetic	Mechanism	Model	Results	Toxicity	Ref.
Au + Ni_80_Fe_20_ (permalloy) 1 μm radius disk-shaped	Dynamic 1 T at 20 Hz rotating	Vortex shaped rotation	In vivo mice glioma	Increased survival	No change in histology No side effects	[50]
pEGFP/p53 conjugated	Static	Gene therapy + magnetofection	In vitro BBB + glioblastoma	Increased induced apoptosis	-	[51]
Aptamer conjugated dextran coated	Alternate 9.55 kA/m at 1 Hz	3D Rotating nanosurgeons	In vitro glioblastoma	Increased induced apoptosis	-	[52]
Octadecyl-quaternized carboxymethyl chitosan	Static 0.5 T	Delivery loaded paclitaxel	In vivo rats glioma	Increased survival Prolonged bioavailability	Reduced side-effects	[53]
Inhibitor of metalloproteinase-1 conjugated	Static 0.8 T	Crossing BBB and regulation of metalloproteinases	In vitro BBB + HIV infection	Recovery in spine density ROS and HIV infection level decrease	Unchanged TEER and cells viability	[54]
Bilayers: Tenofovir + dextran Sulphate + vorinostat	Static 0.08 T 6 h	Crossing BBB and antiretroviral therapy	In vitro BBB + HIV infection	HIV infection level decrease Prolonged bioavailability	Unchanged TEER and cells viability	[55]
Azidothymidine 5′-triphosphate loaded CoFe_2_O_4_@BaTiO_3_	Static 22 Oe/cm 6 h Alternate 66 Oe at 100 Hz 5 min	Crossing BBB and controlled release of antiviral drug	In vitro BBB + HIV infection	Functional and structural integrity of the drug after the release	High cell viability	[56]
Beclin1 siRNA binded CoFe_2_O_4_@BaTiO_3_	Static 0.8 T 3 h	Crossing BBB and regulate autophagy	In vitro BBB + HIV infection	Attenuate HIV-1 replication and viral-induced inflammation	Unchanged TEER and occludin expression	[57]
Morphine antagonist, CTOP conjugated	Static 0.5 T	Crossing BBB and drug delivery	In vitro BBB + HIV infection	Recovery in spine density Prevention of morphine induced apoptosis	High cell viability Unchanged TEER	[59]
PEG shell	Alternate 15 kA/m 500 KHz	Heat-sensitive capsaicin receptor TRPV1 activated by magnetothermal genetic stimulation	In vitro neurons + in vivo mice	On demand activation of neurons in deep nuclei (VTA)	Lower glial activation and macrophage accumulation compared to implant	[60]
CoFe_2_O_4_-BaTiO_3_ GMO coated	Static 3000 Oe/cm Alternate 100 Oe at 0–20 Hz	Crossing and concentrate in brain then modulate neural activity	In vitro + in vivo mice	EEG detectable modulation activity of 1 mV	No toxicity for astrocytes and blood cells in vitro	[61]
Co-ferrite core and Mn-ferrite shell Polymer PMA coated	Alternate 7–30 kA/m at 412–570 KHz	Heat-sensitive capsaicin receptor TRPV1 activated by magnetothermal genetic stimulation	In vitro neurons + in vivo mice	On demand evoked motor cortex ambulation, striatum rotation or freezing	-	[62]
GFP-tagged ferritin	Alternate 23–31 mT at 465 kHz	Heat-sensitive capsaicin receptor TRPV1 activated by magnetothermal genetic stimulation	In vitro neurons + in vivo mice	Glucose-sensing hypothalamus neurons modulate feed behavior	-	[63]
Starch-coated	Static 150 mT	Magnetic force open neuron channels	In vitro neuron	Mechanical opening of N-type mechanosensitive Ca^2+^ channels	Reversibility of opening	[65]
Starch and chitosan coated	Static 150 mT	Magnetic force open neuron channels	In vitro neuron	Mechanical opening of N-type mechanosensitive Ca^2+^ channels	Unchanged cell viability, reversibility of opening	[66]
Ferumoxide-labeled human neural stem cells	Static 0.32 T	Magnetic targeting	In vivo stroke rats	Better targeting and recovery in a stroke model	Unchanged differentiation into neurons or astrocytes	[68,69]
Dextran-coated	Alternate 1–6 A at 0.25–2 Hz 10 min	Osmotin load targeting in hippocampus and delivery	In vitro + in vivo AD rats	Memory improvement Reduced protein accumulation and synaptotoxicity	Unchanged viability No apoptosis No BBB leakage	[70,71]
Oleic acid-coated	3 days	Gene therapy delivery Alpha-Synuclein RNAi Plasmid	In vitro + in vivo PD mice	Motor improvement Reduced neurodegeneration	No organ damage Normal blood test 12 days	[73]
Uncoated Fe_3_O_4_	Alternate 2 h/d for 1 week	Synergic effect of magnetic stimulation and MNPs	In vivo PD rats	Motor improvement/recover feeding behavior Reduced ROS and lesion volume	Normal mitochondrial activity	[74]
